# Can farmers’ digital literacy improve income? Empirical evidence from China

**DOI:** 10.1371/journal.pone.0314804

**Published:** 2024-12-02

**Authors:** Tian Liu, Lan Liao

**Affiliations:** 1 Economic Department, Capital University of Economics and Business, Beijing, China; 2 People’s Bank of China Nanchong Branch, Nanchong, China; Sichuan Agricultural University, CHINA

## Abstract

This paper explore the impacts and mechanisms of digital literacy of farm households on income. The baseline regression of the impact of digital literacy of farm households on household income uses a fixed effects regression model, and the 2SLS regression model is used to address the endogeneity problem present in the model. The findings reveal that improving digital literacy among rural households significantly increases their family income, a result that remains robust even after considering endogeneity issues. Further examination of the mechanisms shows that enhancing digital literacy among rural households significantly improves their information acquisition capabilities and cognitive skills. It also deepens financial services, boosting the usage and engagement of rural households in digital financial activities, thereby enhancing family income levels. Facilitating rural residents’ access to digital skills and tools to ride the digital economic wave, ensuring fair access, and achieving sustainable family income are of paramount significance for rural revitalization. It is also a crucial step in bridging the digital divide and promoting shared prosperity.

## Introduction

As digital rural play a crucial role in promoting rural revitalization and shared prosperity, the increasing influence of digital literacy on rural household income deserves attention. In this context, the stabilizing role of the digital economy in economic development has become increasingly prominent, and digital construction is a strategic choice to seize new opportunities in the era of technological revolution and industrial transformation. Accelerating the pace of building a digital society is not only a necessary requirement for promoting modernization but also a crucial breakthrough in addressing rural issues and a vital path to achieving rural revitalization. According to data from the National Bureau of Statistics, in 2021, the per capita disposable income of rural residents reached 19,831 yuan, while the per capita disposable income of urban residents in the same year was over 47,412 yuan, more than double that of rural residents. It can be seen that the long-term accumulation of a dual urban-rural economic structure has led to significant disparities in social development between urban and rural areas, making it essential to conduct continuous and in-depth research on increasing rural household income.

In 2018, the "Opinions on Implementing the Rural Revitalization Strategy" officially proposed to "vigorously promote the construction of digital rural areas," emphasizing the need to improve the quality of rural livelihoods and bridge the digital divide. Currently, the rapid development of the digital economy is driving profound changes in production methods, lifestyles, and governance. Narrowing the digital divide among rural households, enhancing their digital literacy, and promoting farmers’ "income growth and stability" are paramount in achieving rural revitalization.

Existing literature has not reached a consistent conclusion regarding the impact of digital literacy on income. Krueger found that there is a significant wage premium for using the internet, with internet users earning a wage premium of 10% to 15% compared to non-users [1]. Lee and Kim argued that using the internet at work leads to a significant wage premium primarily due to the substantial increase in individual labor productivity associated with internet use [2]. Atasoy obtained consistent results using U.S. county-level data, particularly highlighting more significant income increases in rural and remote areas [3]. Most studies suggest that digital usage significantly promotes residents’ income. However, some research indicates that this impact is not significant and may even be negative [4, 5]. Possible reasons for these discrepancies include the fact that internet usage exhibits significant technological bias and that increased competition resulting from internet usage can suppress wage growth. It is evident that the impact of digital literacy on income has not yet reached a consensus in academia, and further scientific methods and representative data are still required to investigate this issue [5].

However, existing literature has primarily focused on the impact of digital finance, digital economy on income, and income inequality, with limited attention to the influence of digital literacy on income. For instance, Song *et al*. used China Labor Dynamics Survey (CLDS) data and found that digital finance has a significant positive impact on the agricultural income of rural farmers [6]. Kolade and Owoseni argued that the development of the digital economy promotes the rapid growth of flexible employment, thereby increasing wage income for rural laborers, such as food delivery drivers, ride-sharing drivers, and couriers. However, caution is necessary regarding income inequality resulting from the digitization process [7]. Jiang *et al*. analyzed provincial panel data from 2009 to 2019 and discovered a significant "U-shaped" relationship between the digital economy and urban-rural income disparities [8]. Some literature highlights that digital literacy improves farm household incomes through mechanisms such as health [9], information [10]. This paper takes a micro-level perspective, empirically analyzes the impact of rural households’ digital literacy on family income and income structure, and analyzes the mechanism of digital literacy’s impact on residents’ income from the perspectives of information barriers, human capital, and financial services.

The primary contribution of this paper, in contrast to the existing literature, lies in addressing the lack of a consistent measurement system for digital literacy. Consequently, there is a pressing need to establish a scientific indicator system to explore the impact of digital literacy on income. Digital literacy encompasses an individual’s awareness, attitude, and capacity to utilize digital tools for communication, expression, and social action within specific life contexts [11]. Prior research has frequently relied on binary variables, such as "using a mobile phone or not" and "having internet access or not," to empirically assess the effects of digital usage on income [1]. However, with the ubiquitous adoption of the internet, the proportion of samples lacking internet access or mobile phone usage has become insignificant, thereby reducing the representativeness of these variables. Erstad emphasized that engagement in the digital realm is no longer determined by "having" or "not having" but rather by one’s capabilities [12]. A limited number of recent studies have initiated the use of continuous variables to measure individual digital literacy [9, 10, 13]. This paper constructs a comprehensive indicator evaluation system for rural households’ digital literacy, encompassing five facets: learning, work, entertainment, social interaction, and creativity. This system aims to holistically reflect rural households’ digital literacy levels. We empirically analyze the influence of digital literacy on farmers’ wage income, business income, property income, and transfer income. It is crucial to note that analyzing the impact of digital literacy on total farm household income from a solitary perspective cannot overlook the significance of transfer income. If digital literacy primarily boosts transfer income, it does not necessarily imply an increase in farm household income.

## Theoretical analysis and research hypothesis

With the rapid development of the digital internet, the digital literacy of individual farmers has become a crucial factor influencing family decisions. Specifically, digital literacy includes digital awareness, computational thinking, digital learning and innovation, and digital social responsibility. Theoretically, as human capital in the digital age, digital literacy primarily manifests in the use, processing, and creation of new value through digital technology. Digital technology, in turn, can affect individual decisions by altering the relative income and costs of farmers’ participation in various economic activities. As a form of "technical" human capital, digital literacy enables individual farmers to integrate into the digital society and enjoy digital benefits. In general, digital literacy influences income through the following three channels:

First, the improvement of digital literacy can alleviate information poverty and increase farmers’ income. The development of digital communication technology enriches information acquisition channels, enhancing farmers’ information acquisition capabilities and generating information diffusion effects, thus increasing farmers’ income. Holman studied job quality in Europe from the perspective of employment types and found that internet usage helps reduce information asymmetry in the labor market [14]. Job seekers can promptly and efficiently obtain relevant job information, and the increase in employment opportunities contributes to improving job quality. Dettling found that using the internet for job searches significantly reduces job seekers’ search costs and increases the probability of obtaining employment [15]. Moreover, the information effect redistributes welfare between farmers and intermediaries, with some of the welfare originally obtained by intermediaries being transferred to farmers, ultimately raising the income level of the farmer population [16]. Goyal demonstrated that the introduction of internet kiosks in India providing price information and quality testing significantly increased soybean prices [17]. Based on this, Hypothesis 1 is proposed.

Hypothesis 1: Digital literacy can expand farmers’ information acquisition channels, enhance their information acquisition capabilities, and, by alleviating information poverty, have a promoting effect on family income, thereby increasing farmers’ income. This forms the "digital literacy—information effect—farmers’ income" mechanism.

Second, digital literacy can promote the accumulation of human capital and increase farmers’ income. Digital platforms provide a variety of skill training courses, offering farmers more opportunities to access vocational training. Through online courses, farmers can gain knowledge of their professions, enhance their personal qualities, and improve their professional skills, leading to returns in the form of skill premiums [18]. Aker and Ksoll demonstrated that the acquisition of mobile phone technology and learning how to use it brought about some economic benefits to specific rural populations [19]. Acemoglu *et al*. studied the impact of artificial intelligence on the labor market and found a rapid increase in job vacancies related to artificial intelligence, while recruitment for non-artificial intelligence positions decreased [20]. Furthermore, the development of digital technology has made remote education and online learning gradually become new ways for farmers to access educational resources. The deep integration of digital technology with vocational training has also improved the timeliness of training, enhanced the human capital of workers, and become an important supplement to formal education systems [21]. Based on this, Hypothesis 2 is proposed.

Hypothesis 2: Digital literacy can promote investment in human capital by farmers, through the human capital effect, leading to an increase in family income, thus forming the "digital literacy—human capital effect—farmers’ income" mechanism.

Third, digital literacy can deepen financial services and promote income growth among farmers. The improvement of farmers’ digital literacy allows more farmers to access financial services, alleviating financial constraints for agricultural entrepreneurship and providing diversified wealth opportunities for families. This optimization of resource allocation leads to increased income for farmers. Kass used South African data to empirically analyze that digital literacy is a key factor in building financial resilience [22]. The development of digital technology provides feasible funding for farmer entrepreneurship, increasing farmers’ enthusiasm and possibilities for entrepreneurship. Digital lending offers flexible borrowing and repayment methods, with the entire transaction process, from customer information collection and risk analysis to loan disbursement, being digitized. This digitalization reduces financing costs, effectively mitigates financial constraints during entrepreneurship, provides sustainable financial supply for rural residents [23], and enhances family resilience and resilience [24]. Based on this, Hypothesis 3 is proposed.

Hypothesis 3: Digital literacy can expand the scope of digital financial services and deepen financial services. By mitigating farmers’ financial constraints and broadening wealth opportunities, it has a promoting effect on family income, thereby increasing farmers’ income. This forms the "digital literacy—financial services effect—farmers’ income" mechanism.

## Materials and methods

### Data

This study utilizes data from the 2020 China Family Panel Studies (CFPS), China County Statistical Yearbooks, various provincial and municipal statistical yearbooks, and the Regional Inclusive Finance Index published by Peking University’s Digital Finance Research Center. As this study primarily investigates the impact mechanism of rural households’ digital literacy on family income, only data with rural household registration status are retained. Additionally, any abnormal values, such as those in the explanatory variables like education level, age, gender, health status, and responses like "unknown" or "not applicable," have been removed.

The dependent variable in this study is the per capita family net income of rural residents (*Income*), along with subcategories such as per capita family wage income (*Wage Income*), per capita family operating income (*Operating Income*), per capita family property income (*Property Income*), and per capita family transfer income (*Transfer Income*).

The core explanatory variable is rural household digital literacy. The concept of "digital literacy" was first introduced by Paul Gilster in his work "Digital Literacy" [25]. He believed that digital literacy is not just the technical ability to use digital devices but, more importantly, the application of these skills in daily life. With the rapid development of information and communication technology (ICT), numerous studies have defined the concept of digital literacy. The EU DigEuLit project defines digital literacy as follows: digital literacy refers to an individual’s awareness, attitude, and ability to appropriately use digital tools and facilities to identify, access, manage, integrate, evaluate, analyze, and synthesize digital resources in specific life contexts, construct new knowledge, create media expressions, communicate with others, and engage in constructive social actions, reflecting on this process [26]. As defined by the Cyberspace Administration of China, digital literacy and skills refer to a set of qualities and abilities that digital society citizens should possess, including digital acquisition, production, use, evaluation, interaction, sharing, innovation, security, ethics, and morality. Consequently, this study constructs a comprehensive evaluation system for rural household digital literacy from five perspectives: learning, work, entertainment, social interaction, and creation.

In the CFPS questionnaire, questions are included on the frequency of internet use for learning, work, entertainment, social interaction, and commercial activities. The usage frequency is assigned values from 1 to 7 in ascending order, where higher values indicate higher digital usage frequency and higher digital literacy in households. Due to the CFPS questionnaire assigning values from 1 to 7 with higher values indicating higher household digital usage frequency, for the sake of clarity in interpreting the regression results, we has assigned values from 1 to 7 to represent low to high household digital usage frequency. The arithmetic mean of the five indicators is computed as the comprehensive measure of rural household digital literacy.

There are three categories of control variables: regional economic characteristics, individual characteristics, and dummy variables. The economic characteristics include government financial support for agriculture, the level of agricultural modernization, industrial structure, and market size as regional economic variables. Government financial support for agriculture is measured by the proportion of expenditure on agriculture, forestry, and water affairs to local general public budget expenditure. Agricultural modernization level is measured by the total power of agricultural machinery. Industrial structure is measured by the proportion of value-added by the primary industry to total production value. Market size is measured by total retail sales of social consumer goods, with the natural logarithm of retail sales used in the analysis. Government support for agriculture is measured by the proportion of agricultural and forestry expenditures to local general public budget expenditures. Agricultural modernization is measured by the total power of agricultural machinery. Industrial structure is measured by the proportion of the value added of the primary industry to the total production value. Market size is measured by the total retail sales of consumer goods, which is then transformed using a logarithmic function.

Individual characteristics include the age, gender, education, whether the household head is engaged in agricultural work, ethnicity, marriage, Communist Party members, job type and health status of the household head. Descriptive statistics of the main variables are presented in Table 1. Overall, there is a significant disparity in family income among the survey samples. Wage income varies significantly among households, and family income sources are diverse and differentiated. The average digital literacy among households is 1.77, indicating that the average digital usage frequency among rural households is relatively low, leaving significant room for improvement in rural household digital literacy. Table 1 shows the descriptive statistical analysis.

**Table 1:** Descriptive statistics of key variables.

variables	range	mean	standard deviation
**Dependent variable**			
per capita household income (*Income*)	(0,5660000)	23395.19	76551.09
per capita household wage income (*Wage Income*)	(0,60000)	13908.83	21214.15
per capita household operating income (*Operating Income*)	(0,160500)	330.23	3030.99
per capita household property income (*Property Income*)	(0,5500000)	3121.30	56851.16
per capita household transfer income (*Transfer Income*)	(0,3000000)	2736.17	39457.10
**Core independent variable**			
digital literacy (*Digital*)	(0,7)	1.77	2.23
**Area-specific variables**			
government financial support for agriculture (*Government*)	(0.04,0.19)	0.11	0.04
agricultural modernization (*Agri-Modern*)	(94,10415)	4288.29	3233.12
industrial structure (*Industry*)	(0.32,23.40)	9.51	4.38
market size (*Market*)	(6.6,19.10)	10.02	2.55
**Personal characteristic variables**			
Occupation (*Agri-Work*)	(0,1)	0.57	0.50
Age (*Age*)	(16,93)	49.40	15.03
Gender (*Gender*)	(0,1)	0.53	0 .50
Education (*Education*)	(0,3)	1.22	0.58
Ethnicity (*ethnicity*)	(0,38)	1.65	2.67
Health (*Health*)	(0,5)	3.05	1.53
*Marriage*	(0,1)	0.824	0.381
*Communist Party members(Party)*	(0,1)	0.070	0.255
*Type of work(job)*	(1,5)	2.230	1.435

### Empirical methodology

This paper draws inspiration from the classic Mincer earnings equation [27] and controls for other relevant factors to separately examine the impact of farmers’ digital literacy on household income, with the following econometric model specified.


$$Log(Income_{i})=\alpha_{0}+\alpha_{1}Digital_{i}+\alpha_{2}X_{i}+\varepsilon_{i}$$ (1)

In Eq (1), the dependent variable, *Income_i_*, represents the income level of the family i, and the key explanatory variable, *Digital_i_*, represents the logarithm of the digital literacy of the family i. Control variables are denoted as *X_i_*, including both the regional economic level and individual level. *α* represents the estimated coefficients, and *ε_i_* represents the error term.

It’s worth noting that the bidirectional causality between key variables within the model can lead to endogeneity issues. To address the endogeneity problem, we consider using a Two-Stage Least Squares (2SLS) model. In this 2SLS model, the distance from the household’s location to the central city(*Distance*) and the monthly mobile phone expenses(*Fee*) are used as instrumental variables for digital literacy. The 2SLS model under consideration for addressing the endogeneity issue is as follows:

First Stage:

$$Log(Digital_{i})=\gamma_{0}+\gamma_{1}Distance_{i}+\gamma_{2}Fee+\beta X_{i}+\varepsilon_{i}$$ (2)

Second Stage:

$$Log(Income_{i})=\alpha_{0}+\alpha_{1}Log(Digital_{i})+\beta X_{i}+\varepsilon_{i}$$ (3)

*X_i_* represents the control variables, and *ε_i_* is the random error term. Digital literacy is related to the distance from the household’s location to the city center and the monthly mobile communication fee. We use them as instrumental variables and incorporate them into Eq (2) to re-estimate the impact of digital literacy on household income through instrumental variable regression.

## Estimation results and analysis

### Baseline regression results

The baseline regression results show that digital literacy significantly increases household income (see Table 2). For every one-unit increase in digital literacy, household per capita net income increases by 0.141%. When comparing different types of income, it is found that digital literacy has the greatest impact on household wage income and the lowest impact on property income. On the one hand, digital technology breaks down information asymmetry in rural areas, allowing agricultural information to rapidly penetrate every aspect of production and enabling farmers to optimize land, labor, and capital inputs based on the information they receive, thus improving production and operation efficiency. On the other hand, digital platforms facilitate faster information flow, directly helping farmers connect with demand-side parties, increasing the matching efficiency between land transfer parties, and ultimately enhancing land transfer efficiency.

**Table 2:** The impact of digital literacy on household income.

	(1)	(2)	(3)	(4)	(5)
*VARIABLES*	*Income*	*Wage income*	*Operating income*	*Property income*	*Transfer income*
*Digital*	0.141***	0.387***	0.205***	0.070***	-0.219***
	(26.65)	(19.28)	(9.74)	(4.63)	(-12.06)
*Constant*	38.066**	74.681	0.052	-61.350	-12.682
	(2.05)	(1.26)	(0.00)	(-1.57)	(-0.22)
*Province FE*	YES	YES	YES	YES	YES
*Controls*	YES	YES	YES	YES	YES
*Observations*	8,276	8,276	8,162	8,259	8,101
*R-square*	0.217	0.153	0.238	0.062	0.153

^a^ Robust t-statistics in parentheses, *** p<0.01, ** p<0.05, * p<0.1; the values in parentheses below the coefficients represent the t-values.

### Robustness check

Panel A presents the regression results with the replacement of explanatory variables. In this analysis, digital literacy is calculated by conducting factor analysis on five dimensions: learning, work, entertainment, social, and business. The resulting digital literacy variable is weighted by factor coefficients to represent farmers’ digital literacy. Panel B shows the results using truncated regression. To mitigate the influence of extreme values on the regression results, the data is confined to the 1st to 99th percentile of the original data. Panel C demonstrates the results using the Tobit model, replacing the OLS model. Given that there are many zero values in the household income data, a Tobit model is further employed to estimate the impact of digital literacy on household income while addressing the issue of zero values.

Table 3 presents the results of robustness checks. It can be observed that digital literacy significantly enhances the income level of rural households, which is consistent with the previous estimation results, indicating the robustness of the conclusions drawn in this study.

**Table 3:** Robustness test of the impact of digital literacy on household income.

Panel A: Substitute explanatory variables					
*VARIABLES*	*Income*	*Wage income*	*Operating Income*	*Property Income*	*Transfer Income*
*Digital*	0.427***	1.203***	0.611***	0.207***	-0.683***
	(26.30)	(19.46)	(9.55)	(4.55)	(-12.28)
*Constant*	38.216**	75.926	-0.215	-61.375	-13.681
	(2.06)	(1.28)	(-0.00)	(-1.57)	(-0.24)
*City FE*	YES	YES	YES	YES	YES
*Observations*	8,276	8,276	8,162	8,259	8,101
*Controls*	*YES*	*YES*	*YES*	*YES*	*YES*
*R-square*	0.215	0.154	0.237	0.062	0.154
Panel B:Excluding the influence of outliers					
*VARIABLES*	*Income*	*Wage income*	*Operating Income*	*Property Income*	*Transfer Income*
*Digital*	0.136***	0.386***	0.204***	0.065***	-0.220***
	(29.48)	(19.26)	(9.71)	(4.39)	(-12.23)
*Constant*	49.688***	74.659	-0.597	-66.285*	-8.048
	(3.25)	(1.26)	(-0.01)	(-1.70)	(-0.14)
*Observations*	8,276	8,276	8,162	8,259	8,101
*Controls*	*YES*	*YES*	*YES*	*YES*	*YES*
*City FE*	*YES*	*YES*	*YES*	*YES*	*YES*
*R-square*	0.261	0.152	0.239	0.062	0.156
Panel C: Substitution regression method					
*VARIABLES*	*Income*	*Wage income*	*Operating Income*	*Property Income*	*Transfer Income*
*Digital*	0.141***	0.476***	0.429***	0.070***	-0.219***
	(25.29)	(17.18)	(10.28)	(5.07)	(-12.40)
*Constant*	37.936**	93.598	43.442	-61.307	-12.687
	(2.26)	(1.12)	(0.34)	(-1.48)	(-0.23)
*Observations*	8,276	8,276	8,162	8,259	8,101
*Controls*	*YES*	*YES*	*YES*	*YES*	*YES*
*Province FE*	*YES*	*YES*	*YES*	*YES*	*YES*

^a^ Robust t-statistics in parentheses, *** p<0.01, ** p<0.05, * p<0.1; the values in parentheses below the coefficients represent the t-values.

### Endogeneity test

The chosen indicator for measuring digital literacy in this study is the frequency of rural households using the internet for learning, entertainment, socializing, consumption, and business activities. It is acknowledged that there might be a two-way causal relationship between digital literacy and household income. Generally, higher-income individuals tend to use the internet more frequently for consumption and entertainment, and vice versa. To deal with the potential endogeneity issue, the study employs instrumental variable (IV) methods and utilizes a 2SLS (Two-Stage Least Squares) model.

In general, when the government is involved in the construction of digital infrastructure and the provision of digital services, it usually spreads from the city center to the surrounding areas. Therefore, the farther an area is from the city center, the lower the level of digital development in that area. The frequency and likelihood of residents in that area enjoying digital services are also lower. However, the income level of residents is related to the overall economic development of the local area and is not strongly influenced by the distance from the city center. The income gap between residents living in the suburbs of the city and those living in the city center is not significant within the city.

Furthermore, residents who use digital services more frequently tend to have higher mobile phone expenses, which are relatively fixed and only account for a small portion of their consumption expenditures. These expenses lack income elasticity and do not significantly increase with an increase in monthly income.

In terms of instrumental variable selection, this paper chose the driving distance from the households’ location to the city center of this city and the monthly household mobile communication expenses as instrumental variables for measuring the digital literacy of households.

Table 4 presents the first-stage estimates of the 2SLS regression. From the table, it can be observed that rural households’ digital literacy is significantly negatively correlated with the distance from their location to the city center. In other words, the farther a household’s location is from the city center, the lower the level of digital development in that area, and consequently, the lower the digital literacy of rural residents in that area. There is a significant positive correlation between monthly phone expenses and digital literacy. As monthly phone expenses for households increase, their digital literacy also increases.

**Table 4:** First stage of IV estimation.

VARIABLES	Distance	Fee
Digital	-0.002***	0.013***
	(-4.74)	(17.41)
Unidentifiability test: Kleibergen-Paap rk LM statistic	617.19	
Weak instrument test: Cragg-Donald Wald F statistic	511.47	
Weak instrument test: Kleibergen-Paap Wald rk F statistic	161.92	
Overidentification test: Hansen-J statistic	24.946	
Observations	7,721	

The Kleibergen-Paap LM test for instrument validity significantly rejects the null hypothesis that there is an identification problem in the first stage. Additionally, both the Cragg-Donald Wald test and the Kleibergen-Paap Wald rk test for weak instrument validity far exceed the critical value of 10, indicating the absence of weak instrument problems. The results of the Hasman-J overidentification test also indicate no overidentification problem with the instruments.

Table 5 presents the estimation results after considering endogeneity. The regression results indicate that rural households’ digital literacy significantly improves family income. It is worth noting that, using instrumental variable estimation, the impact of digital literacy on rural households’ per capita net income, wage income, and operating income becomes larger, indicating that the baseline regression underestimated the influence of digital literacy on rural household income. Furthermore, the impact of digital literacy on per capita property income is not significant, suggesting that digital literacy has a limited influence on rental income from land or other production assets, as well as income from housing rentals for rural households.

**Table 5:** IV estimation results.

	(1)	(2)	(3)	(4)	(5)
VARIABLES	Income	Wage income	Operating Income	Property Income	Transfer Income
Digital	0.291***	0.501***	0.347***	0.010	-0.584***
	(18.04)	(7.66)	(5.27)	(0.23)	(-10.11)
Constant	69.953***	99.270	19.059	-67.265	-23.616
	(4.20)	(1.63)	(0.33)	(-1.64)	(-0.38)
Observations	7,721	7,721	7,621	7,704	7,556
Province FE	YES	YES	YES	YES	YES
Controls	YES	YES	YES	YES	YES
R-squared	0.155	0.138	0.227	0.060	0.111

^a^ Robust t-statistics in parentheses, *** p<0.01, ** p<0.05, * p<0.1; the values in parentheses below the coefficients represent the t-values.

## Mechanism test

This article analyzes the mechanism through which rural household digital literacy influences family income from three perspectives: information acquisition, human capital, and digital financial services. We use the "importance of using the internet to obtain information" as a proxy variable for information acquisition capability (*Message*). The level of family financial service usage (*Finance*) is measured by the number of financial services such as savings, loans, and insurance used by the household. Additionally, according to traditional human capital theory, human capital is essentially equivalent to cognitive ability, while the new human capital theory considers human capital to include both cognitive and non-cognitive abilities [28]. Due to data availability, this study primarily examines the cognitive ability component of human capital, using the scores obtained by respondents in digital test questions (*Math*) and word test questions (*Word*) as proxy variables for cognitive ability.

Columns (1) to (2) in Table 6 explore the "digital literacy—information effect—household income" mechanism. The regression results indicate that for every 1% increase in digital literacy, the information acquisition ability of rural households increases by 0.491 units. For every 1-unit increase in information acquisition ability, the total income of rural households increases by 0.034%. Information poverty is one of the important reasons for economic poverty. Information poverty and economic poverty are positively correlated. Poverty in information sources leads to resource scarcity, and poverty in information acquisition ability leads to technological economic poverty. Poverty in the ability to interpret and use information leads to knowledge-based economic poverty. Enhancing rural digital literacy helps households bridge the gap in traditional information resources, break environmental constraints, broaden horizons, access timely and effective market information, and alleviate resource-related economic poverty.

**Table 6:** The impact mechanisms of digital literacy.

	(1)	(2)	(3)	(4)	(5)	(6)	(7)	(8)
VARIABLES	Message	Income	Math	Income	Word	Income	Finance	Income
Digital	0.491***	0.119***	0.742***	0.103***	1.636***	0.094***	0.054***	0.130***
	(73.02)	(20.40)	(29.26)	(18.54)	(33.73)	(16.28)	(13.70)	(27.87)
Message		0.034***						
		(4.55)						
Math				0.023***				
				(7.55)				
Word						0.015***		
						(10.10)		
Service								0.114***
								(7.74)
Constant	44.869**	50.426***	-99.460	45.139***	-459.641***	48.029***	2.009	49.460***
	(1.98)	(3.28)	(-1.37)	(2.59)	(-3.07)	(2.74)	(0.19)	(3.27)
Controls	YES	YES	YES	YES	YES	YES	YES	YES
Province FE	YES	YES	YES	YES	YES	YES	YES	YES
Observations	8,238	8,238	6,412	6,412	6,213	6,213	8,276	8,276
R-squared	0.445	0.264	0.227	0.227	0.232	0.236	0.117	0.266

^a^ Robust t-statistics in parentheses, *** p<0.01, ** p<0.05, * p<0.1; the values in parentheses below the coefficients represent the t-values.

Columns (3) to (6) in Table 6 investigate the "digital literacy—human capital effect—household income" mechanism. The regression results show a significant positive correlation between digital literacy and the cognitive ability of rural household members. By promoting the accumulation of human capital through the enhancement of cognitive abilities, digital literacy significantly increases the total income of rural households. The accumulation of human capital is a key driver of economic growth driven by technological progress and innovation. It can enhance labor productivity, promote business innovation and technological progress, and ultimately increase residents’ income levels by improving occupational levels. Improving rural digital literacy helps households compensate for the lack of offline educational resources through online learning materials, training courses, and discussion forums, enhancing individual cognitive abilities, promoting the accumulation of personal human capital, improving work capabilities and marginal labor output, and thereby increasing the income levels of rural households.

Columns (7) to (8) in Table 6 explore the "digital literacy—financial services effect—household income" mechanism. The regression results show that for every 1% reduction in secondary digital literacy, rural households’ access to financial services increases by 0.54 units. For every 1-unit increase in family financial services, rural household total income increases by 0.114%. Digital inclusive finance provides small credit projects, savings, insurance, and other transaction services, which are beneficial for poverty reduction. Compared to traditional inclusive finance, it can more effectively increase the income levels of rural residents [29]. Improving rural digital literacy contributes to the dissemination of inclusive financial and microfinance services in rural areas, reducing the credit and financial service costs for households, expanding production scale, and improving production efficiency.

To further validate the robustness of the impact mechanisms, this study conducted robustness tests on the mechanisms using the Sobel and Bootstrap methods. Table 7 reports the results of these tests, and it can be observed that all Sobel test values for the intermediate variables reject the null hypothesis. Additionally, the indirect effects of the variables in the Bootstrap tests do not contain the value 0 within the 95% confidence intervals. Therefore, the improvement of rural household digital literacy significantly enhances their information acquisition capabilities, cognitive abilities, and deepens their use of financial services. This, in turn, increases the income levels of rural households. In other words, there exist the impact mechanisms of "digital literacy—information effect—farmer income," "digital literacy—human capital effect—farmer income," and "digital literacy—financial services effect—farmer income."

**Table 7:** Sobel and Bootstrap tests for the impact mechanisms of digital literacy.

Variables	Sobel	Effect	value	SE	[95% Conf. Interval](BC)	
*Message*	4.551	Indirect Effect	0.017	0.004	0.010	0.024
		Direct Effect	0.119	0.005	0.108	0.130
*Math*	7.461	Indirect Effect	0.017	0.002	0.012	0.021
		Direct Effect	0.103	0.005	0.093	0.114
*Word*	9.891	Indirect Effect	0.024	0.003	0.020	0.029
		Direct Effect	0.094	0.005	0.082	0.105
*Finance*	6.688	Indirect Effect	0.006***	0.001	0.004	0.008
		Direct Effect	0.130***	0.005	0.120	0.139

## Conclusion and implications

This paper based on the CFPS household database, has examined the impact of digital literacy on rural household income. It has demonstrated how digital literacy affects household income through the channels of information acquisition, human capital, and financial services. This paper not only enriches the theoretical framework concerning the relationship between digital literacy and economic development but also provides significant theoretical insights for the formulation of policies aimed at increasing rural household income and promoting common prosperity. The study has found a significant positive correlation between rural household digital literacy and income. This relationship remains robust even when considering alternative explanatory variables, regression methods, and remove outliers regression. After accounting for endogeneity issues, it has been determined that for every 1% increase in digital literacy, per capita net income of rural households increases by 0.291%. This underscores the importance of digital literacy in increasing income for farmers.

These findings have important policy implications for increasing rural incomes and promoting shared prosperity. The following policy recommendations are proposed: (1) Promote Rural Digitalization: The government and relevant agencies should take various measures to enhance rural digital literacy and address skill gaps. This includes investments in digital infrastructure and the establishment of long-term mechanisms to support digital literacy and skill development. (2) Encourage Social Engagement: Encourage active participation from various sectors of society in initiatives aimed at improving digital literacy and skills for all. Attract social capital and corporate involvement in digital rural development and digital skills training activities, creating a diversified support system. (3) Boost Farmers’ Initiative: As rural digitalization continues to progress, the urban-rural digital divide is narrowing. However, disparities in usage still exist, particularly in areas such as information access, online operations, and communication compared to urban residents. It is essential to encourage farmers to actively participate in digital skills training, enhancing their individual digital literacy and skills, and increasing their sense of empowerment in the digital economy era.

## Limitations and future research

The limitations of this study are primarily centered around several key aspects. First, while the measurement system for digital literacy is comprehensive, it may still fall short in fully capturing the nuances and complexities of digital literacy across different individuals and groups. Variations in how individuals perceive and use digital tools may not be entirely reflected in the evaluation system employed. Second, the impact of digital literacy on income could be influenced by numerous factors beyond the scope of this study, such as economic policies, educational backgrounds, and technological advancements. These external factors may have affected the results but were not accounted for in the study’s design. Third, due to the use of cross-sectional data in analyzing the impact of farmers’ digital literacy on household income, the study was unable to capture the dynamic characteristics and potential longitudinal relationships between farmers’ digital literacy and their household income. As a result, while the study provides valuable insights into the current state of farmers’ digital literacy and its association with household income, it does not offer a comprehensive understanding of how these variables evolve and interact over time.

Future research directions in this field can follow several promising avenues. First, there is a need to refine and enhance the measurement system for digital literacy to better capture the nuanced differences and complexities across diverse demographic and socio-economic groups. This could involve developing more sophisticated indicators and incorporating qualitative methods to complement quantitative data. Second, future studies should investigate the interplay between digital literacy and income in the context of various external factors, such as economic policies, educational backgrounds, and technological advancements. Considering these factors would provide a more comprehensive understanding of the multifaceted relationships involved. Additionally, there is potential for longitudinal studies to track the evolution of digital literacy skills over time and their long-term impact on individual income and career trajectories. Such research would offer valuable insights into the development of digital literacy and its sustained influence on economic outcomes.
